# Clinicopathologic characteristics and prognosis of Borrmann type IV gastric cancer: a meta-analysis

**DOI:** 10.1186/s12957-016-0805-9

**Published:** 2016-02-24

**Authors:** Yifan Luo, Peng Gao, Yongxi Song, Jingxu Sun, Xuanzhang Huang, Junhua Zhao, Bin Ma, Yuan Li, Zhenning Wang

**Affiliations:** Department of Surgical Oncology and General Surgery, First Hospital of China Medical University, Shenyang City, Liaoning Province People’s Republic of China

**Keywords:** Gastric cancer, Borrmann type IV, Clinicopathologic characteristic, Prognosis, Meta-analysis

## Abstract

**Background:**

The clinicopathologic features and surgical treatment strategy of Borrmann type IV (B-4) gastric cancer remains controversial. This meta-analysis was conducted to evaluate the clinicopathologic features of patients with B-4 gastric cancer and to assess whether or not non-curative resection improved prognosis.

**Methods:**

PubMed and Embase were searched for relevant articles. Statistical analysis was performed using RevMan (version 5.2). The odds ratio (OR), risk ratio (RR), hazard ratio (HR) with 95 % confidence interval (CI), and weighted average of median survival times were calculated as effect values.

**Results:**

Fifteen studies were included. Compared with Borrmann type “others” (B-O), B-4 had a higher incidence of poorly differentiated carcinoma (OR = 4.92; 95 % CI = 3.10–7.83; *P* < 0.01), lymph node metastases (OR = 2.13; 95 % CI = 1.88–2.41; *P* < 0.01), peritoneal metastases (OR = 3.91; 95 % CI = 3.37–4.54; *P* < 0.01), serosal invasion (OR = 3.66; 95 % CI = 2.91–4.60; *P* < 0.01), and lymphatic invasion (OR = 1.39; 95 % CI = 1.02–1.91; *P* = 0.04). B-4 patients with non-curative resection were associated with a worse survival rate (HR = 2.83; 95 % CI = 2.35–3.40; *P* < 0.01) than patients with curative resection; however, B-4 patients with non-curative resection had a better survival rate (1-year: RR = 0.70, 95 % CI = 0.63–0.77; *P* < 0.01; 2-year: RR = 0.90, 95 % CI = 0.85–0.94; *P* < 0.01) than patients with non-resection.

**Conclusions:**

Our meta-analysis indicated that B-4 patients were associated with poor tumor differentiation, lymph node metastases, peritoneal metastases, serosal invasion, lymphatic invasion, and prognosis. Curative resection may increase the survival rate for B-4 patients. If it is not possible to perform a curative resection, a non-curative resection may improve the prognosis.

## Background

Gastric cancer is a common malignant disease and remains the third most frequent cause of cancer deaths worldwide [1, 2]. The classification of advanced gastric cancer according to Borrmann’s criteria is presently accepted by many surgeons, endoscopists, and radiologists worldwide. According to this classification, Borrmann type IV (B-4) gastric cancer is defined as a lesion which diffusely infiltrates to the gastric wall without ulceration or distinct elevation [3]. The incidence of B-4 gastric cancer is approximately 10–20 % of all gastric cancer [4]. Several studies have analyzed the clinicopathologic features of patients with B-4 gastric cancer, but controversy still remains with respect to the incidence of hepatic metastases and vascular invasion [5–8]. In addition, B-4 gastric cancer usually has a poor prognosis; the 5-year survival rate after gastrectomy has been reported to be approximately 30 % [8, 9]. Surgical resection is the most suitable treatment for gastric cancer [10], but the surgical treatment strategy for B-4 patients is controversial [11–14]. Some investigations have reported that non-curative resection may improve the prognosis of patients with B-4 gastric cancer [13]. At the same time, it was reported by another group that there was no statistical difference between the prognosis of patients with non-curative resection and those with non-resection [14]. Some researchers even believe that B-4 gastric carcinoma is not surgically curable based on the poor outcomes after surgery [11, 12]. The purposes of our meta-analysis were to compare the clinicopathologic characteristics (e.g., gender ratio, pathologic type, tumor metastases, and invasion) between B-4 gastric cancer and Borrmann type “others” (B-O) and to evaluate the effectiveness of surgical treatment for patients with B-4 gastric cancer.

## Methods

### Search strategy

Two investigators (Yifan Luo and Peng Gao) performed independent searches of the electronic databases (PubMed and Embase) from inception to January 2015. The search strategy included the keywords “Borrmann” and “gastric cancer” and the strategy was changed according to different requirements for each database. Both published and unpublished articles were included, and no language restriction was applied. The reference lists of all selected studies were further searched to identify other additional pertinent articles.

### Inclusion and exclusion criteria

All observational and experimental studies that evaluated survival of patients with B-4 gastric cancer treated by non-curative resection were considered, and the studies that compared the clinicopathologic characteristics of B-4 and B-O gastric carcinomas were included in the present study. Articles without full text and data needed that could not be acquired from the authors were excluded. In addition, letters to the editor without useful data, case reports, and editorials were excluded. If the same authors reported multiple investigations conducted during the same period, we only utilized the most complete reports in the present study.

### Data extraction

Two independent researchers extracted data from each study using a predefined table. The following information was extracted from the included study: author, publication time, country, sample size, clinicopathologic characteristics, and major end point. The pathologic type was separated into well (tubular and papillary adenocarcinomas)- and poorly differentiated (poorly differentiated adenocarcinoma, mucinous carcinoma, and signet-ring cell carcinoma) types. If the article did not provide the hazard ratio (HR) for overall survival, we used Engauge Digitizer 4.1 software to distinguish the Kaplan–Meier curves and extract the HRs of overall survival.

### Quality assessment for included studies

The quality of each included study was independently evaluated using the Newcastle–Ottawa Scale [15] by two investigators, with scores ≥5 indicating high quality.

### Statistical analysis

Statistical analysis was performed with RevMan (version 5.2; Cochrane Collaboration). The odds ratio (OR) and risk ratio (RR) with 95 % confidence interval (CI) were used for the analysis of dichotomous data, and the weighted average of the median survival times were used for continuous data. The HR and 95 % CI for the overall survival were calculated with the method reported by Tierney et al. [16]. We assessed the heterogeneity between studies using the chi-square test, which indicates the presence of significant heterogeneity at a *P* value <0.05. At the same time, *I*^2^ was used to assess heterogeneity. An *I*^2^ > 50 % was considered to be statistically significant. For outcomes in which significant heterogeneity was observed, a random effects model was used; otherwise, a fixed effects meta-analysis was conducted. The assessment of publication bias was evaluated using the funnel plot.

## Results

### The included literature and methodologic quality

**Fig. 1:**
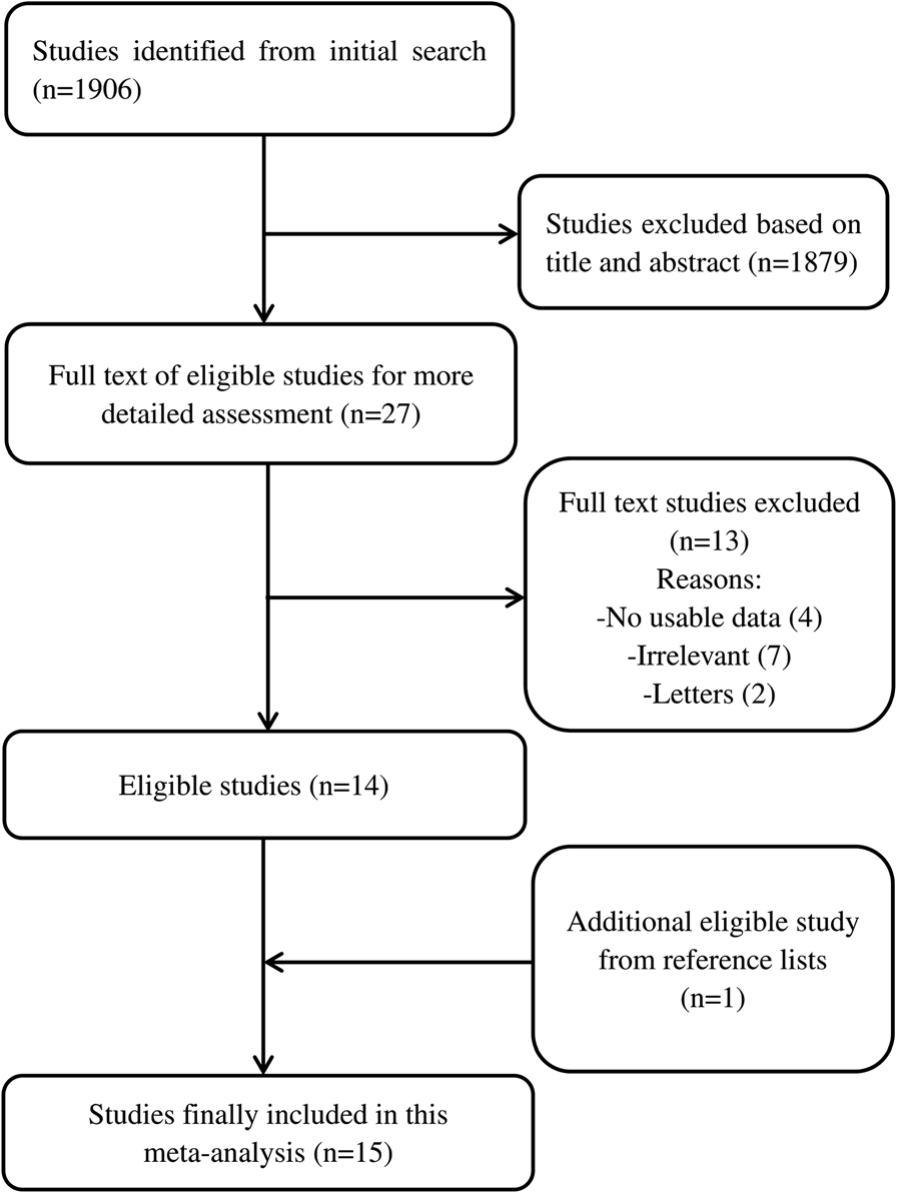
Flow diagram of the studies search and selection

A flow diagram of the selection process is shown in Fig. 1. The initial search identified 1906 studies for the meta-analysis; 1879 studies were excluded after a review of the titles and abstracts. After full text assessment of the 27 eligible studies, 15 were included in this meta-analysis according to our inclusion and exclusion criteria. All included studies were retrospective. The main characteristics of each included study are listed in Table 1. The quality of the included studies was assessed according to the Newcastle–Ottawa Scale; most of the included studies were high quality based on the scores (>5).

**Table 1:** Characteristics of included studies

Author	Year	Country	B-4/B-O (patient number)	B-4 incidence (%)	B-4 with non-curative resection				B-4 with non-resection				NOS
					Patient number	Median survival time (month)	1-year survival	2-year survival	Patient number	Median survival time (month)	1-year survival	2-year survival	
Maehara [6]	1992	Japan	194/919	17.4	NA	NA	NA	NA	NA	NA	NA	NA	6
Kitamura [14]	1995	Japan	102/563	15.3	50	11.1	NA	NA	13	6.2	NA	NA	5
Tanigawa [17]	1998	Japan	33/169	16.3	NA	NA	NA	NA	NA	NA	NA	NA	6
Otsuji [12]	1999	Japan	150/698	17.7	NA	NA	NA	NA	NA	NA	NA	NA	5
Yokota [19]	1999	Japan	88/309	22.2	NA	NA	NA	NA	NA	NA	NA	NA	6
Kodera [22]	2001	Japan	70/0	NA	31	9.7	35.48 %	12.90 %	11	8	27.27 %	9.09 %	6
Chen [5]	2002	China	103/604	14.6	NA	NA	NA	NA	NA	NA	NA	NA	5
Kim [18]	2002	Korea	199/1607	11	NA	NA	NA	NA	NA	NA	NA	NA	5
Yook [13]	2005	Korea	370/3693	9.1	51	11	47.06 %	13.73 %	90	7	26.67 %	11.10 %	6
Nashimoto [23]	2007	Japan	770/8474	8.3	254	9	37.80 %	14.57 %	116	4.33	7.76 %	0.86 %	5
An [8]	2008	Korea	555/3636	13.2	NA	NA	NA	NA	NA	NA	NA	NA	4
Li [9]	2009	China	517/3449	13	NA	NA	NA	NA	NA	NA	NA	NA	5
Accetta [20]	2011	Brazil	123/836	12.8	16	10	NA	13.00 %	55	3	NA	0	5
Gao [21]	2011	China	118/0	NA	32	11.6	40.60 %	6.20 %	19	6.1	15.80 %	0	5
Ma [7]	2012	China	139/754	15.6	NA	NA	NA	NA	NA	NA	NA	NA	7

*NOS* Newcastle–Ottawa Scale, *B-4* Borrmann type IV, *B-O* Borrmann type “others”, *NA* not applicable

### Clinicopathologic characteristics and prognosis

**Fig. 2:**
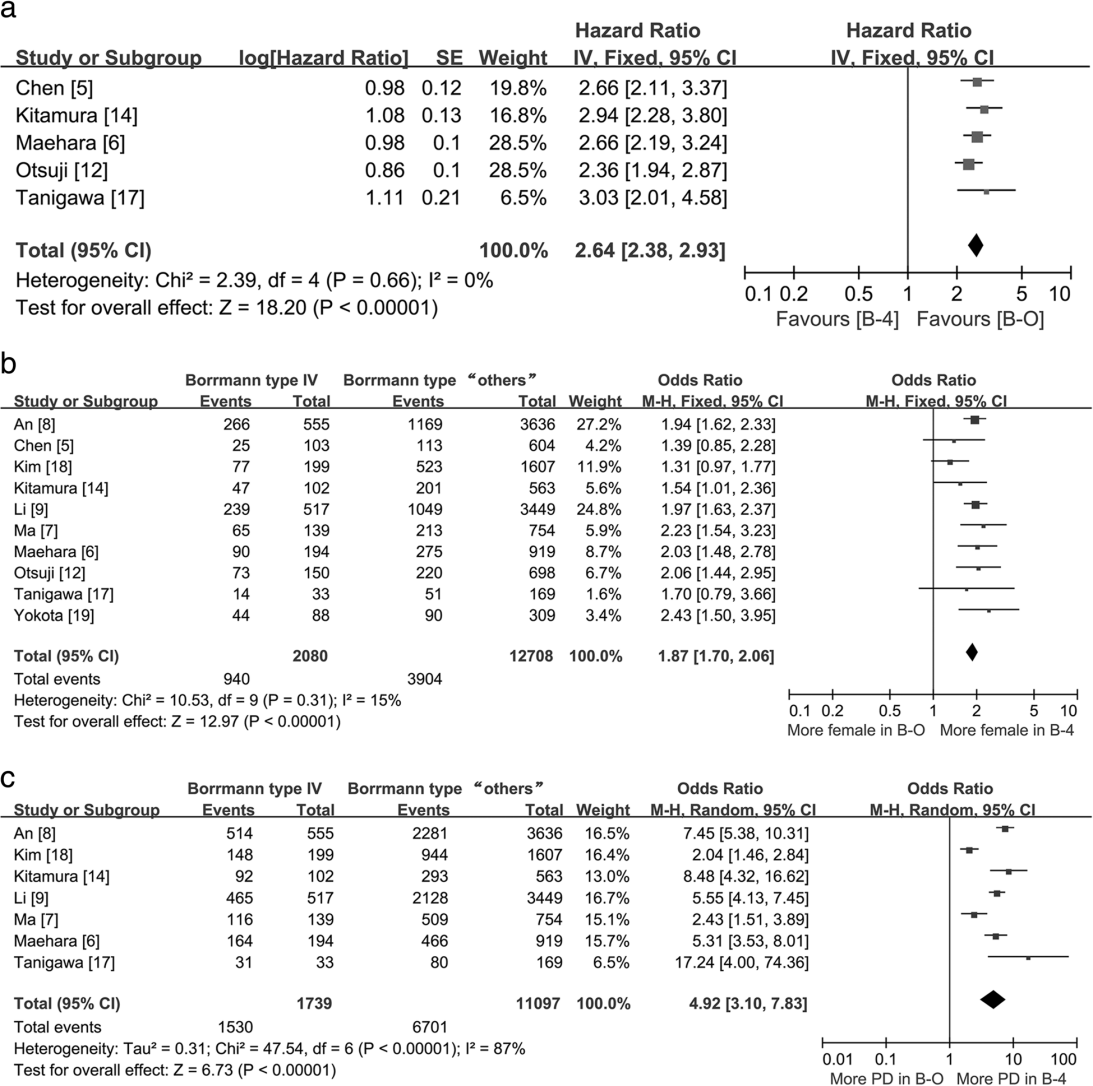
Forest plot displaying the results of meta-analysis. **a** Hazard ratio for overall survival of patients with B-4 or B-O. **b** Odds ratio for gender ratio. **c** Odds ratio for pathologic type (*B-4* Borrmann type IV, *B-O* Borrmann type “others”, *PD* poorly differentiated carcinoma)

We demonstrated that patients with B-4 carcinoma had significantly worse survival rates than patients with other types of carcinomas (HR = 2.64; 95 % CI = 2.38–2.93; *P* < 0.01; Fig. 2a). Ten studies provided data regarding the gender ratio. The female-to-male ratio in the B-4 group was significantly higher than that in the B-O group (0.82 [940/1140] vs. 0.44 [3904/8804]; pooled OR = 1.87; 95 % CI = 1.70–2.06; *P* < 0.01; Fig. 2b). In the pathologic type subset, seven studies involving 12,836 patients were divided into two principal subgroups (well- and poorly differentiated). In the poorly differentiated group, the proportion of B-4 patients was significantly higher than the B-O patients (87.98 % [1530/1739] vs. 60.39 % [6701/11,097]; pooled OR = 4.92; 95 % CI = 3.10–7.83; *P* < 0.01; Fig. 2c).

**Fig. 3:**
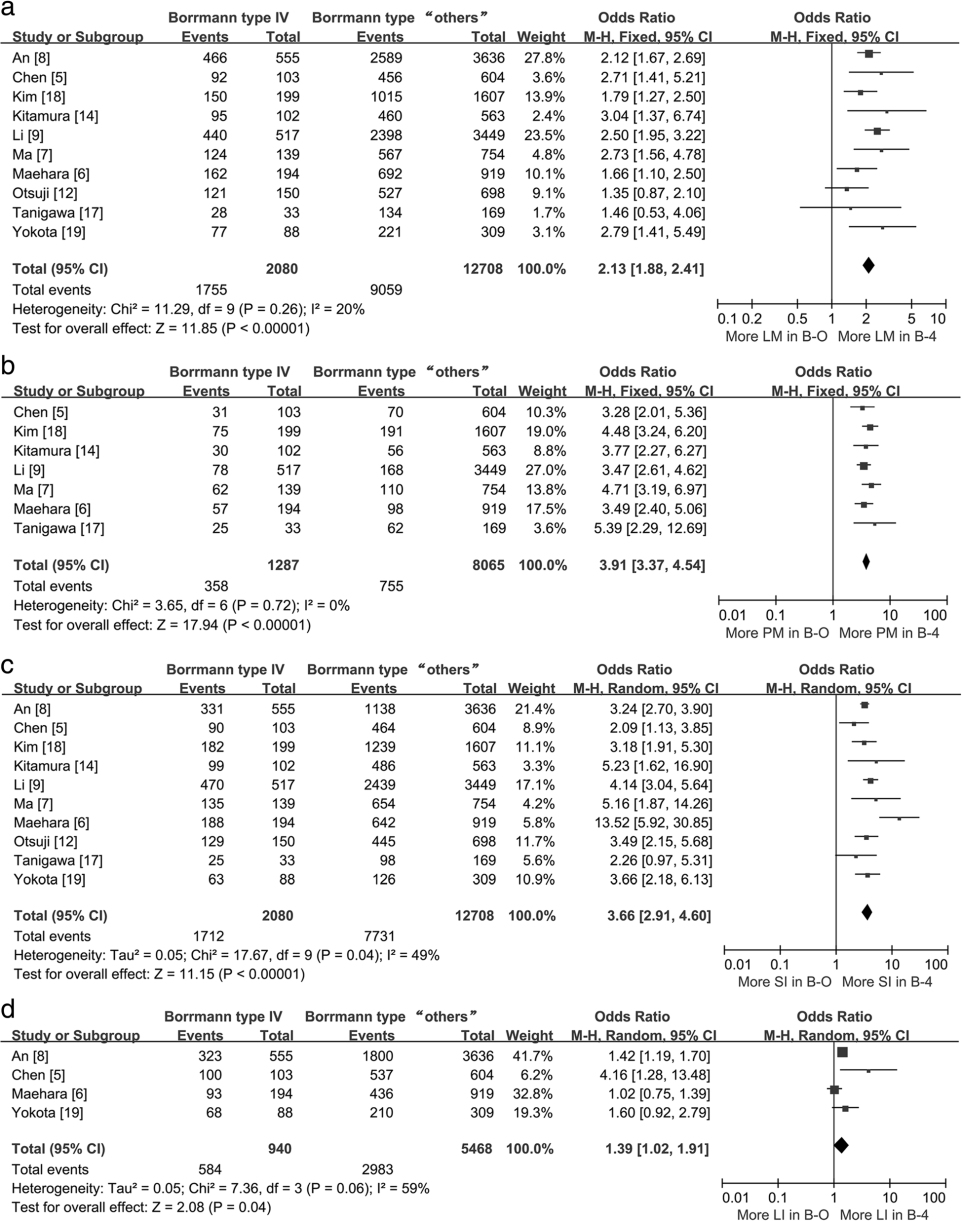
Forest plot displaying the results of meta-analysis. **a** Odds ratio for lymph node metastases. **b** Odds ratio for peritoneal metastases. **c** Odds ratio for serosal invasion. **d** Odds ratio for lymphatic invasion (*B-4* Borrmann type IV, *B-O* Borrmann type “others”, *LM* lymph node metastases, *PM* peritoneal metastases, *SI* serosal invasion, *LI* lymphatic invasion)

**Fig. 4:**
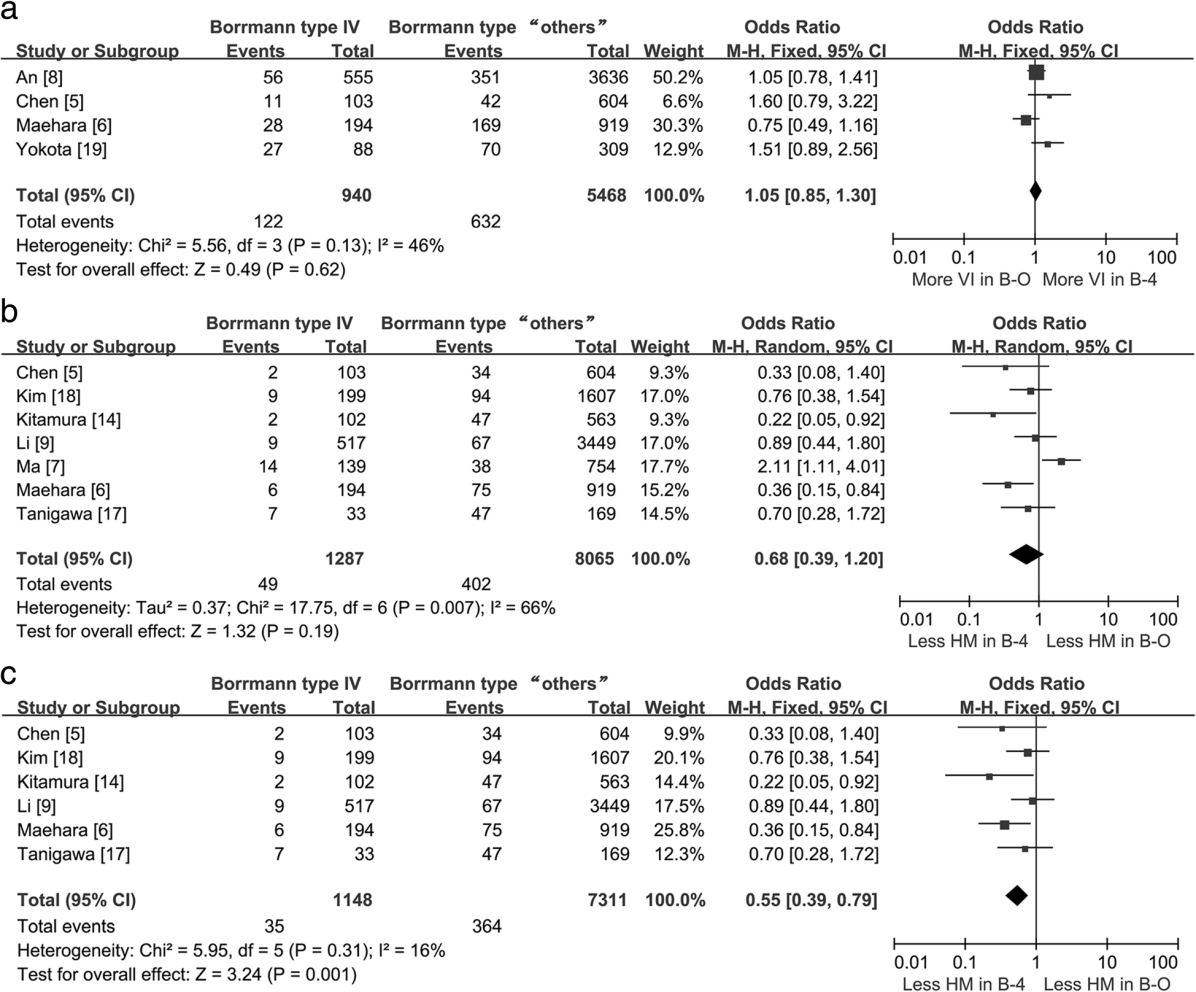
Forest plot displaying the results of meta-analysis. **a** Odds ratio for vascular invasion. **b** Odds ratio for hepatic metastases. **c** Sensitivity analysis of odds ratio for hepatic metastases (*B-4* Borrmann type IV, *B-O* Borrmann type “others”, *VI* vascular invasion, *HM* hepatic metastases)

In addition, ten trials [5–9, 12, 14, 17–19] showed data on tumor metastases and invasion, which we distributed into six subsets (lymph node metastases, hepatic metastases, peritoneal metastases, lymphatic invasion, serosal invasion, and vascular invasion). The different types of metastases and invasion patterns resulted in different outcomes. A higher ratio of B-4 patients with lymph node metastases (84.38 % [1755/2080] vs. 71.29 % [9059/12,708]; pooled OR = 2.13; 95 % CI = 1.88–2.41; *P* < 0.01; Fig. 3a), peritoneal metastases (27.82 % [358/1287] vs. 9.36 % [755/8065]; pooled OR = 3.91; 95 % CI = 3.37–4.54; *P* < 0.01; Fig. 3b), serosal invasion (82.31 % [1712/2080] vs. 60.84 % [7731/12,708]; pooled OR = 3.66; 95 % CI = 2.91–4.60; *P* < 0.01; Fig. 3c), and lymphatic invasion (62.13 % [584/940] vs. 54.55 % [2983/5468]; pooled OR = 1.39; 95 % CI = 1.02–1.91; *P* = 0.04; Fig. 3d) was shown compared with B-O patients; however, there were no statistically significant differences in vascular invasion (pooled OR = 1.05; 95 % CI = 0.85–1.30; *P* = 0.62; Fig. 4a) and hepatic metastases (3.81 % [49/1287] vs. 4.98 % [402/8065]; pooled OR = 0.68; 95 % CI: 0.39–1.20; *P* = 0.19; Fig. 4b). Sensitivity analysis was performed by omitting each individual study. When we eliminated the Ma study [7], B-4 patients had a lower incidence of hepatic metastases (3.05 % [35/1148] vs. 4.98 % [364/7311]; pooled OR = 0.55; 95 % CI = 0.39–0.79; *P* < 0.01; Fig. 4c), and statistically significant heterogeneity between the remaining studies was not detected (*P* = 0.31, *I*^2^ = 16 %).

### Efficiency of surgical treatment

**Fig. 5:**
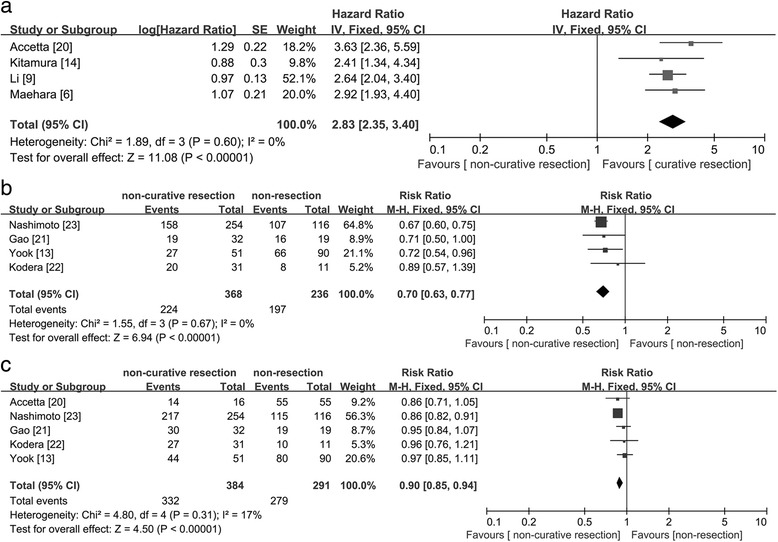
Forest plot displaying the results of meta-analysis. **a**: Hazard ratio for overall survival of patients with Borrmann type IV gastric cancer received non-curative resection or curative resection. **b**: Risk ratio for 1-year survival of patients with Borrmann type IV gastric cancer received non-curative resection or non-resection. **c**: Risk ratio for 2-year survival of patients with Borrmann type IV gastric cancer received non-curative resection or non-resection

A quantitative meta-analysis using the data from four studies revealed that non-curative resection compared with curative resection in B-4 patients was associated with a worse survival rate (HR = 2.83; 95 % CI = 2.35–3.40; *P* < 0.01; Fig. 5a). Six studies reported median survival times for B-4 patients after non-curative resection or non-resection [13, 14, 20–23]. In these studies, 434 patients underwent non-curative resection and 304 patients underwent non-resections, such as an exploratory laparotomy. In the non-curative resection group, the weighted average of the median survival time was 9.8 months, whereas the weighted average of the median survival time was 5.2 months in the other group. Five studies provided data regarding the 1- and 2-year survival rates for B-4 patients. The 1 (RR = 0.70, 95 % CI = 0.63–0.77; *P* < 0.01; Fig. 5b)- and 2-year survival rates (RR = 0.90, 95 % CI = 0.85–0.94; *P* < 0.01; Fig. 5c) in the non-curative resection group had a better prognosis than in the non-resection group. Publication bias was examined by the funnel plot. There was no evidence of publication bias among these comparisons.

## Discussion

Although advances in diagnostic techniques and treatment methods have improved overall treatment outcomes for gastric cancer patients, the prognosis of B-4 gastric cancer remains poor. Patients with B-4 gastric cancer are always in an advanced stage when diagnosed, and some authorities attribute this finding to the special clinicopathologic characteristics of the tumor [13]; however, the clinicopathologic characteristics and surgical treatment strategy of B-4 patients is always in dispute. Based on our present meta-analysis, patients with B-4 gastric cancer had a higher female-to-male ratio, more poorly differentiated carcinomas, more lymph node metastases, more peritoneal metastases, more serosal invasion, more lymphatic invasion, and a poorer prognosis. It has been reported that patients with a poorly differentiated pathologic type of gastric cancer are characterized by lymph node metastases, serosal invasion, peritoneal dissemination, and advanced stage [24]. Some investigators have reported that B-4 gastric cancer predominates in females and in undifferentiated histology, invades the serosal surface, involves lymph nodes more frequently, and has a high incidence of peritoneal dissemination [6, 9, 13, 14]. Indeed, our results are consistent with these studies.

According to our findings, patients with B-4 gastric cancer had a lower incidence of hepatic metastases, but the difference was not statistically significant. Further sensitivity analysis revealed that the Ma study [7] was the primary cause of the heterogeneity. After eliminating the Ma study [7], the incidence of hepatic metastases was significantly lower in patients with B-4 gastric cancer than patients with B-O, and there was no statistically significant heterogeneity between the remaining studies. In the hepatic metastases comparison, we found that patients in the Ma study [7] had a higher serosal invasion incidence than other studies. The high proportion of patients with an advanced stage might be the source of the heterogeneity. Maehara [25] suggested that hepatic metastases were more frequent in patients with Borrmann type 2 and 3 gastric cancer. Adacki [26] reported that most poorly differentiated carcinomas are hypovascular and vascular irregularity often exists. We reasoned that B-4 gastric cancer rarely develops blood-borne metastases to the liver might due to the large proportion of poorly differentiated carcinomas.

The optimal treatment of patients with B-4 gastric cancer remains a matter of debate. In 1989, Aranha [11] concluded that B-4 gastric carcinoma is not a surgically curable disease because of the poor post-operative survival; however, most investigators believe that the prognosis is significantly worse in B-4 patients who undergo a non-curative resection than a curative resection [6, 7, 9, 14, 18]; our results are consistent with their conclusion (HR = 2.83; 95 % CI = 2.35–3.40; *P* < 0.01). This difference may be attributed to the advances in various diagnostic and treatment modalities in recent decades. In Japan, some researchers suggested that aggressive extended surgery, such as a left upper abdominal exenteration plus Appleby’s method (LUAE + Apl), could improve the survival of patients with B-4 stage III gastric cancer [27, 28]. At present, most physicians do not recommend LUAE + Apl because it is not effective for patients with stage IV gastric cancer and may cause severe post-operative complications. Besides, some researchers believe that extended lymphadenectomy should be performed if patient’s clinical condition allowed, because it may allow a better assessment of nodal stations and decrease the chance of stage worsening [20].

Moreover, there is some controversy regarding the role of non-curative resection for survival. Kodera [22] reported that such an aggressive surgical attitude is likely to prove futile for B-4 gastric cancer patients. Yook [13] demonstrated that non-curative resection may lengthen the survival time in B-4 patients with peritoneal dissemination. We found that the 1-year survival rate in the non-curative resection group had a significantly better prognosis than in the non-resection group (RR = 0.70, 95 % CI = 0.63–0.77; *P* < 0.01). Although the difference in the 2-year survival rate between the two groups was smaller (RR = 0.90, 95 % CI = 0.85–0.94; *P* < 0.01), statistical differences still existed. Because the patients without curative resection do not usually survive 2 years, we could not compare the 3- or 5-year survival between the non-resection and non-curative resection groups. Based on our research, the weighted average of the median survival time in the non-curative resection group (9.8 months) was longer than in the non-resection group (5.2 months). This result showed that patients with non-curative resection might have a beneficial survival compared to patients with non-resection, and non-curative resection may improve the quality of life of the patients through relief of symptoms, such as bleeding, strictures, pain, or malnutrition [23]. It has also been suggested that it is not necessary to obtain a negative surgical margin at the time of a non-curative resection because patients with negative and positive resection margins of the gastrectomy with peritoneal disseminations had similar survival rates [13]; some researchers believe that for B-4 patients with peritoneal disseminations but without passage disturbance, non-curative resection should be replaced by chemotherapy [23]. However, more research into this issue is warranted before a final conclusion can be drawn. A randomized controlled trial (RCT) is designed to evaluate the superiority of non-curative resection followed by chemotherapy to chemotherapy alone in terms of overall survival [29]. We look forward to the results of this clinical trial.

Certainly, there were several limitations in our study. First, none of the studies we used in this meta-analysis were RCT studies. Patients were not randomized to non-curative resection or non-resection groups. As a result, we could not ignore the possibility that the non-curative resection group had a better prognosis than the non-resection group due to the difference in patient status; however, in the absence of RCT studies, performing a meta-analysis of retrospective studies, which represents the best evidence available, is necessary and helpful. Second, the sample sizes of some included studies were relatively small and may have weakened the statistical power. Third, with the exception of one study from Brazil, most of our included studies were from Asian countries; thus, the conclusions might not apply to cases outside of Asia, and the results should be further confirmed by studies involving more countries.

## Conclusions

In conclusion, our meta-analysis demonstrated that B-4 gastric cancer patients had a higher female-to-male ratio, more poorly differentiated carcinomas, more lymph node metastases, more peritoneal metastases, more serosal invasion, more lymphatic invasion, and a poorer prognosis. Curative resection of B-4 gastric cancer may increase the survival rate if detected at an early stage. Even if it is not possible to perform curative resection, the effort required to perform non-curative resection may improve the prognosis of patients with B-4 gastric cancer. In the future, all of our results need further confirmation based on well-designed multi-center RCTs from more countries.

## Abbreviations

B-4: Borrmann type IV
B-O: Borrmann type “others”
CI: confidence interval
HR: hazard ratio
LUAE + Apl: left upper abdominal exenteration plus Appleby’s method
OR: odds ratio
RCT: randomized controlled trial
RR: risk ratio
